# Expression of Thymic Stromal Lymphopoietin in Immune-Related Dermatoses

**DOI:** 10.1155/2022/9242383

**Published:** 2022-08-21

**Authors:** Si-Zhe Li, Xin-Xing Jin, Yin Shan, Hong-Zhong Jin, Ya-Gang Zuo

**Affiliations:** ^1^Department of Dermatology, State Key Laboratory of Complex Severe and Rare Diseases, National Clinical Research Center for Dermatologic and Immunologic Diseases, Translational Medicine Center, Peking Union Medical College Hospital, Chinese Academy of Medical Sciences and Peking Union Medical College, Beijing, China; ^2^Department of Dermatology, The First Affiliated Hospital, Zhejiang University School of Medicine, Hangzhou, Zhejiang, China; ^3^Graduate Student of Hebei North University, Zhangjiakou, Hebei, China

## Abstract

Thymic stromal lymphopoietin (TSLP), long known to be involved in Th2 response, is also implicated in multiple inflammatory dermatoses and cancers. The purpose of this study was to improve our understanding of the expression of TSLP in the skin of those dermatoses. Lesional specimens of representative immune-related dermatoses, including lichen planus (LP), discoid lupus erythematosus (DLE), eczema, bullous pemphigoid (BP), psoriasis vulgaris (PsV), sarcoidosis, and mycosis fungoides (MF), were retrospectively collected and analyzed by immunohistochemistry. Morphologically, TSLP was extensively expressed in the epidermis of each dermatosis, but the expression was weak in specimens of DLE. In a semiquantitative analysis, TSLP was significantly expressed in the epidermis in LP, BP, eczema, PsV, sarcoidosis, and MF. TSLP expression was higher in the stratum spinosum in LP, eczema, BP, PsV, and MF and higher in the stratum basale in sarcoidosis and PsV. Moreover, we found positive TSLP staining in the dermal infiltrating inflammatory cells of BP, PsV, and sarcoidosis. Our observation of TSLP in different inflammatory dermatoses might provide a novel understanding of TSLP in the mechanism of diseases with distinctly different immune response patterns and suggest a potential novel therapeutic target of those diseases.

## 1. Introduction

Thymic stromal lymphopoietin (TSLP), a type of epithelial-derived cytokine, was originally identified as a factor promoting B cell proliferation and development [1, 2]. Subsequently, TSLP was found to induce dendritic cells (DCs) or directly act on innate lymphoid cells and CD4+ T cells and to promote the immune responses of helper T type (Th) 2 cells [2, 3]. Furthermore, previous studies have demonstrated that TSLP promotes Th17 cell differentiation under Th2 polarizing conditions [4] and acts on neutrophils to enhance *S. aureus* killing in the innate immune response [5]. Hematopoietic progenitor cells, basophils, eosinophils, mast cells, monocytes/macrophages, and platelets also express TSLP receptors (TSLPR) and thus could be activated by TSLP [3, 6].

Initially, research about TSLP mainly focused on allergic disorders, such as allergic asthma, atopic dermatitis (AD), allergic rhinitis, eosinophilic esophagitis, and food allergies [2, 7, 8]. It was gradually revealed that TSLP is extensively involved in autoimmune diseases and cancers [3]. Moreover, expression of TSLP was found to participate in many inflammatory dermatoses, such as psoriasis vulgaris (PsV) [9, 10], bullous pemphigoid (BP) [11], and alopecia areata [12]. Those diseases distinctly differ in clinical manifestations, pathology, and immune response patterns.

Combining clinical and histopathologic phenotypes with immunology and molecular genetics, noncommunicable inflammatory dermatoses could be classified into six immune response patterns [13]. PsV is a classic psoriatic pattern dermatosis and regards as a Th1/Th17-induced inflammatory disease [13, 14]. Eczema is a common inflammatory dermatosis with widely diverse aetiologies and is considered one of the type 2 immune diseases. BP, the most common autoimmune blister disease, is mainly induced by pathogenic autoantibodies and the Th2 response. TSLP expression was elevated in sera and lesions of patients with PsV and BP [9–11, 15]. But the previous studies have not revealed the specific site of skin lesions that TSLP expressed in those dermatoses. Lichen planus (LP), as a typical lichenoid pattern dermatosis, is primarily induced by cytotoxic CD8^+^ T cells attacking the keratinocytes. Previous research has reported that TSLP expression is elevated in the oral epithelium and sera of patients with oral LP [16, 17]. Sarcoidosis is a typical noninfectious granulomatous pattern disease, characterized by granulomas and fibrosis. Previous studies have shown that the TSLP level in the bronchoalveolar lavage fluid of pulmonary sarcoidosis is not higher than that of normal controls [18]. Thus far, no studies have investigated TSLP expressed in LP and cutaneous sarcoidosis. In conclusion, the involvement of TSLP in multiple dermatoses with different features is notable.

To better understand the exact location and pattern of TSLP expression in the skin of patients suffering from different dermatoses, we analyzed by immunohistochemistry retrospective samples of representative immune-related dermatoses, including LP, discoid lupus erythematosus (DLE), PsV, BP, eczema, sarcoidosis, and mycosis fungoides (MF).

## 2. Materials and Methods

### 2.1. Specimen Selection

The dermatopathology database of the Department of Dermatology at Peking Union Medical College Hospital (Beijing) was queried to identify samples consistent with LP, LE, eczema, BP, PsV, sarcoidosis, and MF. Healthy controls (HC) for stains were recruited from normal surrounding skin in patients with nevi, cysts, or seborrheic keratosis. This study was performed in line with the principles of the Declaration of Helsinki (as revised in 2013). Approval was granted by the Ethical Review Committee of Peking Union Medical College Hospital (ZS-1735). Potential participants who had received systematic glucocorticoids, immunosuppressants, biologics, or long-term topical treatment were excluded from the study. The criteria for specimen selection are shown in Table 1.

### 2.2. Immunohistochemistry

Paraffin sections were deparaffinized and blocked with 3% hydrogen peroxide for 15 min, then incubated with goat serum solution for 1 h at room temperature. Next, samples were incubated with rabbit anti-human TSLP (ProteinTech Group, Rosemont, IL, USA) overnight at 4°C, followed by incubation with HRP-conjugated AffiniPure goat anti-rabbit IgG (ProteinTech Group, Rosemont, IL, USA). The specimens were then stained with DAB (Solarbio, Beijing, China), followed by washing and counterstaining with hematoxylin (Solarbio, Beijing, China).

### 2.3. Specimen Grading

For quantitative image analysis, the slides were scanned using a NanoZoomer 2.0-RS (Hamamatsu, Japan), and five visual fields were selected randomly for each slide. Each section was graded semiquantitatively on a scale of 0–3 (0: negative, 1: mild, 2: moderate, and 3: strong) blindly assessed by two dermatologists in five randomly selected visual fields at high (400X) magnification of scanned sections. The section whose average grade is greater than one was considered a positive case. The gradient of the stratum basale and the stratum spinosum was mathematically calculated by subtracting the stratum basale score from that of the stratum spinosum.

### 2.4. Statistical Analysis

The grades of TSLP expression in the stratum basale and the stratum spinosum were presented as means ± standard deviations. A one-way ANOVA and Dunnett's test (the HC group was considered the control group) were used to determine the significance of grade. One-group Student's *t*-test was used to determine the significance of the gradient of the stratum basale and the stratum spinosum. A one-way ANOVA was used to assess general differences among each dermatosis group, and Bonferroni adjustment was used to assess differences between each dermatosis group pair. The grade correlation of TSLP expression between the stratum basale and the stratum spinosum was evaluated using the Pearson correlation test. Statistical significance was assigned at *p* < 0.05. SAS 9.4 (SAS Institute, Cary, NC, USA) was used for statistical analyses. The Prism 7 software (GraphPad Software, Inc, La Jolla, CA, USA) was used to generate statistical graphs.

## 3. Results

### 3.1. TSLP Was Expressed in the Epidermis of Inflammatory Dermatoses

**Figure 1:**
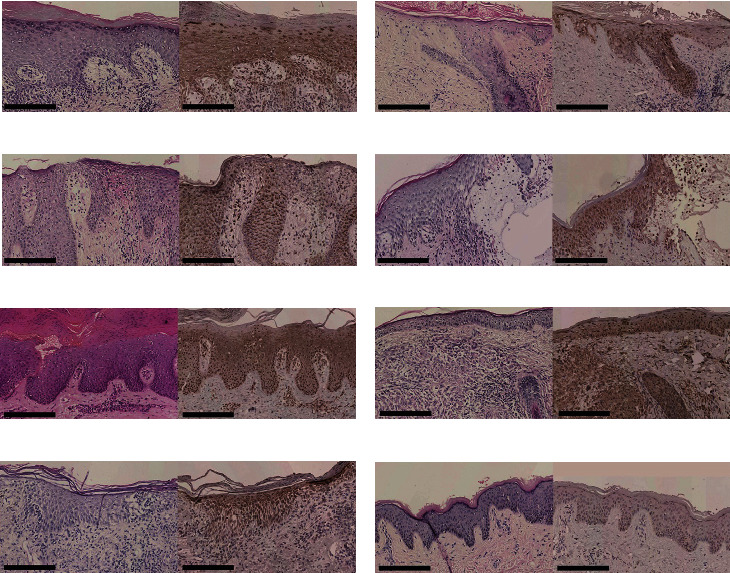
TSLP was expressed in the epidermis of all skin samples: (a) LP, (b) DLE, (c) eczema, (d) BP, (e) PsV, (f) sarcoidosis, (g) MF, and (h) HC. Original magnification: ×200, black scale bar = 200 *μ*m.

A total of 76 specimens, composed of LP (*n* = 10), DLE (*n* = 10), eczema (*n* = 10), BP (*n* = 10), PsV (*n* = 11), sarcoidosis (*n* = 10), MF (*n* = 10), and HC (*n* = 5), were collected for our investigation (Table 2). Morphologically, TSLP was distinctly expressed in the epidermis of LP, BP, eczema, PsV, sarcoidosis, and MF specimens, while in the epidermis with DLE, TSLP-positive keratinocytes could also be observed focally, not as widespread as other dermatoses in our investigation. There were a fewer lightly stained positive cells in the epidermis of HC (Figure 1).

**Figure 2:**
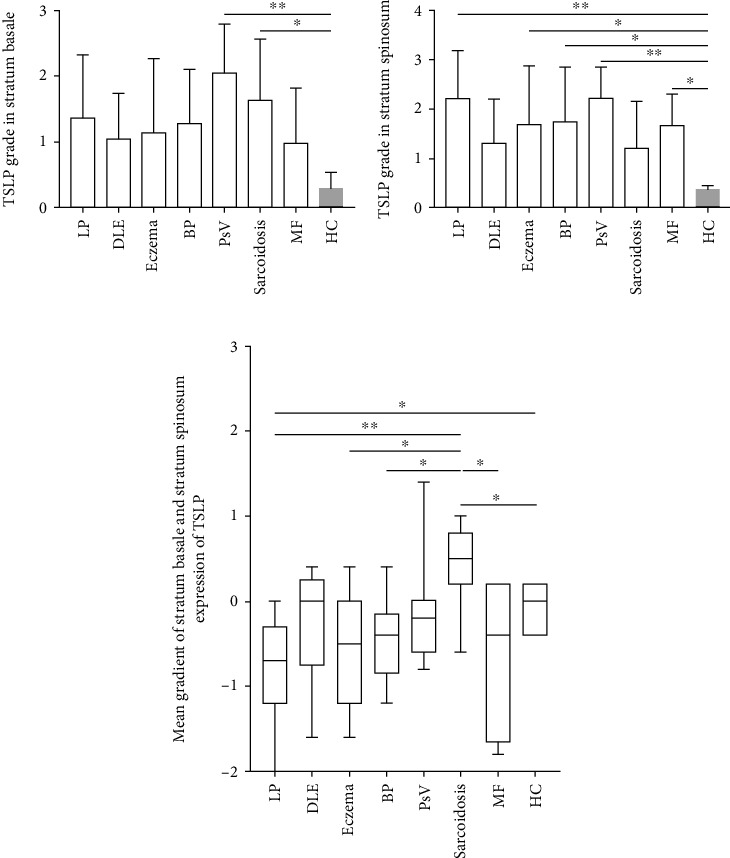
TSLP was significantly expressed in the epidermis in LP, BP, eczema, PsV, sarcoidosis, and MF and nonsignificantly expressed in DLE, in a semiquantitative analysis. (a) TSLP was significantly expressed in the stratum basale of the epidermis with PsV and sarcoidosis specimens. (b) TSLP was significantly expressed in the stratum spinosum of LP, eczema, BP, PsV, and MF. (c) TSLP expression in the stratum basale was significantly higher than that in the stratum spinosum in sarcoidosis specimens and was significantly lower in the stratum spinosum in LP specimens, but not significant in other dermatoses. The mean gradient of stratum basale and stratum spinosum expression of TSLP in sarcoidosis was different from in LP, eczema, BP, and MF, respectively. The score of each section was graded semiqualitatively on a scale of 0–3 (0: negative, 1: mild, 2: moderate, and 3: strong) blindly by two dermatologists in five randomly visual fields at high power field (HPF, ×400 magnification) of scanned sections. The gradient of the stratum basale and the stratum spinosum was mathematically calculated by subtracting the stratum basale score from stratum spinosum. *p* values were determined by the one-group Student *t*-test. Significant differences are noted between the groups: n.s.: not significantly different; ^∗^*p* < 0.05; ^∗∗^*p* < 0.01.

Positive rates of TSLP expression in the epidermis in each group are presented in Table 3, and mean grades of TSLP expression in the stratum basale and the stratum spinosum are presented in Figure 2. Compared to that in HC, TSLP was significantly expressed semiquantitatively in the stratum basale of the epidermis with PsV (*p* = 0.002) and sarcoidosis (*p* =0.025) specimens and not significantly expressed in LP (*p* = 0.102), DLE (*p* = 0.360), eczema (*p* = 0.253), BP (*p* = 0.145), and MF (*p* = 0.436) (Figure 2(a)). Similarly, TSLP was significantly expressed in the stratum spinosum of the epidermis in LP (*p* = 0.002), BP (*p* = 0.031), PsV (*p* = 0.002), eczema (*p* = 0.041), and MF (*p* = 0.046) specimens and not significantly expressed in DLE (*p* = 0.217) and sarcoidosis (*p* = 0.309) (Figure 2(b)). In the epidermis of DLE, the semiquantitative score of TSLP expression was not significant.

To further investigate the diversity of TSLP expression among these dermatoses, we compared the grade between every two groups. There were no significant differences in TSLP expression in both the stratum spinosum and the stratum basale grades among these dermatosis groups, which means TSLP expression did not significantly differ between each disease group overall.

### 3.2. The Differences of TSLP Expression between the Stratum Basale and the Stratum Spinosum

Morphologically, there were no apparent differences in TSLP stain intensity between the stratum basale and the stratum spinosum. However, mean semiquantitative grades in the stratum spinosum were significantly higher than those in the stratum basale in LP (*p* = 0.022) specimens and lower than those in the stratum basale in sarcoidosis (*p* = 0.019) specimens. The differences of TSLP expression between the stratum basale and the stratum spinosum in other dermatoses were not significant (Figure 2(c), DLE *p* = 0.584, eczema *p* = 0.153, BP *p* = 0.146, PsV *p* = 0.777, and MF *p* = 0.173).

Moreover, to further investigate the gradients of the stratum basale and the stratum spinosum expression among dermatoses, we compared it between every two groups. The gradients of the stratum basale and the stratum spinosum expression of TSLP in sarcoidosis were different from LP (*p* = 0.009), eczema (*p* = 0.030), BP (*p* = 0.027), and MF (*p* = 0.023), respectively. The differences of gradients between other dermatoses were not significant (Figure 2(c)).

### 3.3. The Correlations of TSLP Expression between the Stratum Basale and the Stratum Spinosum

Generally, there was a significantly positive correlation in TSLP expression between the stratum spinosum and the stratum basale in all specimens (*r* = 0.713, *p* < 0.001). Similar correlations could also be found in LP (*r* = 0.654, *p* = 0.040), DLE (*r* = 0.673, *p* = 0.033), eczema (*r* = 0.851, *p* = 0.002), BP (*r* = 0.904, *p* < 0.001), PsV (*r* = 0.618, *p* = 0.043), and sarcoidosis (*r* = 0.886, *p* < 0.001) specimens, whereas in MF (*r* = 0.305, *p* = 0.391) and HC (*r* = −0.25, *p* = 0.685) specimens, this correlation was not significant (Table 4).

### 3.4. TSLP Was Also Expressed in the Dermis of BP, PsV, and Sarcoidosis

**Figure 3:**
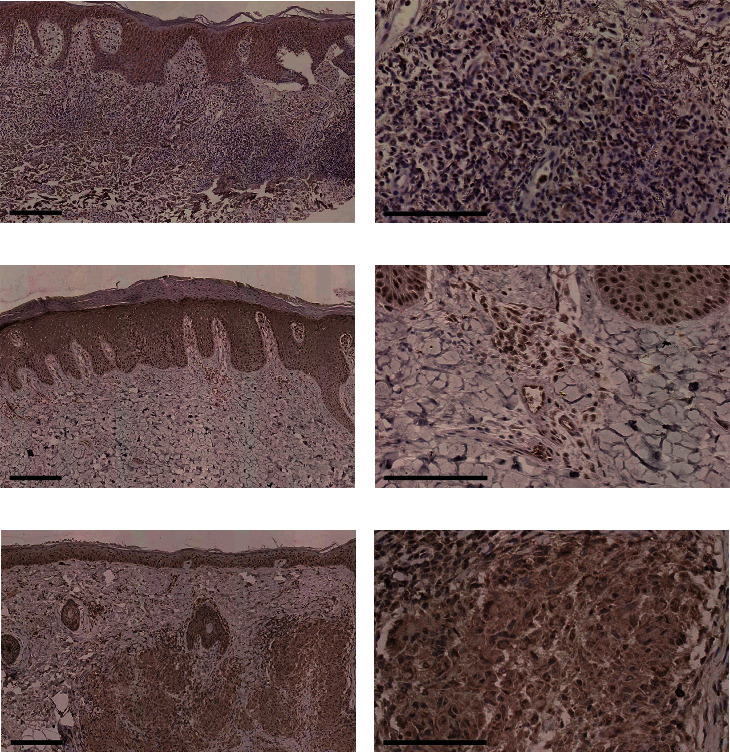
TSLP expressed in infiltrating inflammatory cells in the dermis in BP, PsV, and sarcoidosis. TSLP expressed in the superficial dermis inflammatory cells in BP in (a) and (b), in the superficial perivascular inflammatory cells in PsV in (c) and (d), and in the epithelioid cells in sarcoidosis in (e) and (f). Original magnification: ×100 in (a), (c), and (e), black scale bar = 200 *μ*m; ×400 in (b), (d), and (f), black scale bar = 100 *μ*m.

Finally, we found positive TSLP staining in the dermal infiltrating inflammatory cells in BP, PsV, and sarcoidosis. In BP, TSLP-positive cells scattered among abundant inflammatory cells (Figures 3(a) and 3(b)). In PsV, TSLP was expressed in the dermal superficial perivascular inflammatory cells and endothelial cells (Figures 3(c) and 3(d)). In sarcoidosis, TSLP was expressed in epithelioid cells in “naked” granulomas (Figures 3(e) and 3(f)). Regardless of the extent of inflammatory cell infiltrating into the dermis, we did not observe predominant TSLP expression in other dermatoses specimens.

## 4. Discussion

In our study, we investigated the lesional expression of TSLP in representative dermatoses with different immune patterns. Morphologically, we found TSLP expressed in each dermatosis to some extent. Using semiquantitative analysis, except for DLE, TSLP was expressed in the stratum basale or the stratum spinosum in those dermatoses, and the difference between each dermatosis was not generally significant. Besides, TSLP expression in the stratum spinosum is significantly higher than that in the stratum basale of LP specimens and significantly lower than that in sarcoidosis specimens. Furthermore, the correlations of TSLP expression between the stratum basale and the stratum spinosum were significant, except for MF and HC. In addition, we found that TSLP was also expressed in the dermal infiltrating inflammation cells in BP, PsV, and sarcoidosis. Collectively, our data showed that the expression of TSLP is elevated in the skin of inflammatory dermatosis and may be involved in their pathogenesis.

TSLP is a classic Th2-related cytokine and can induce DC-promoted naïve T cell differentiating to Th2 cells [19]. It could also directly activate CD4^+^ T cells, mast cells, basophils, and eosinophils to promote Th2 immune response [3, 19]. In our study, we found elevated TSLP expression in the epidermis of eczema, BP, and MF, all of which are typical Th2-dominant dermatoses [13, 20]. This result is consistent with previous studies and confirms the relationship of TSLP and Th2 immune response [15, 21–25].

PsV is a Th1/Th17-induced inflammatory disease [13, 14]. However, in our study, we found that TSLP expression was also elevated in the epidermis of PsV. Similar to our finding, previous research demonstrated that the levels of TSLP were elevated in lesions and serum of PsV patients and paralleled to the severity of disease [9, 26, 27]. However, in lesions of a murine model and PsV patients, the TSLP levels rose after being treated with topical vitamin D3 analogs calcipotriol, and it was conducted that calcipotriol could induce TSLP expression, suppress Th1/Th17, and enhance Th2 [28, 29]. It may be due to complex reasons that TSLP plays quite opposite roles in PsV. On the one hand, TSLP is a pleiotropic cytokine that can promote two different groups of DCs, one inducing expression of interferon-*γ*, IL-17A, and IL-22, and the other expressing IL-4, IL-5, IL-9, IL-13, and IL-10 [30]. TSLP was also found to have different immunomodulatory functions in different inflammatory environments [9]. Moreover, some investigators demonstrated that the activity of TSLP was heavily dependent on the TSLP receptors rather than TSLP [27]. Thus, TSLP may also promote the Th1/Th17 immune response in PsV to some extent. On the other hand, those variable results may be caused by heterogeneity of PsV. PsV patients could be divided into two clusters; in one of the clusters, Th2-related gene, including TSLP, was inducted significantly higher in lesions of patients than those present in atopic dermatitis [31]. Therefore, TSLP may promote PsV through Th-2 immune response.

In this study, TSLP was significantly expressed in the epidermis of LP. A similar observation has been documented by Valdebran et al. [24]. Other available reports referred to the relationship between oral LP and TSLP. Sun et al. [16] suggested that TSLP is involved in oral LP by activating the CD8^+^ T cell. Other researches imply that TSLP and other Th2-related chemokines were involved in the pathogenesis [17, 32], which does not support the opinion expressed in a previous study that LP is a Th1 immune response [13]. Similar to PsV, multiple functions of TSLP and heterogeneity of LP might account for the results.

Growing evidence shows that Th1, Th2, and Th17 are associated with the activity and severity of systematic lupus erythematosus (SLE) [33]. Correlation between the TSLP signal pathway and SLE was identified in a previous study [34]. Previous study demonstrated that expression of TSLP in sera and lesions did not significantly increase in SLE patients, compared to HC [35]. Likewise, Soumelis et al. [36] also failed to find TSLP expression increasing in lesions of disseminated lupus erythematosus patient. In our experiment, TSLP-positive keratinocytes could be observed focally in the epidermis of DLE morphologically, but mean grades of TSLP expression in the epidermis did not significantly increase as assessed by the semiquantitative analysis.

Thus far, no studies about the relationship between cutaneous sarcoidosis and TSLP have been reported yet, although one study about idiopathic pulmonary fibrosis showed that TSLP level in the bronchoalveolar lavage fluid of pulmonary sarcoidosis was not higher than that of normal controls [18]. In our study, we found TSLP was significantly expressed in the epidermis of sarcoidosis and the dermal epithelioid cells in “naked” granulomas, which implies that TSLP may be secreted by both keratinocytes and epithelioid cells in cutaneous sarcoidosis. Moreover, it may also account for the high level of TSLP in the stratum basale.

Regarding BP and PsV, the dermal infiltrating inflammatory cells were also positive for TSLP staining, which indicates that TSLP was expressed not only by keratinocytes but also by the infiltrating inflammatory cells in BP and PsV. This observation has not been reported previously, and the specific mechanism is still unknown. Nevertheless, TSLP was also expressed by mast cells, CD163+ macrophage cells, and endothelial cells in other immune-related dermatoses [35, 37, 38]. In addition, TSLP expression in the dermis might be related to a higher expression of TSLP in the stratum basale than in the stratum spinosum in BP and PsV specimens, although the differences were not significant.

Although our study showed no differences of TSLP expression between the stratum basale and the stratum spinosum morphologically, TSLP expression is significantly more intense in the stratum spinosum in LP, which is not consistent with a previous report [24]. By reviewing the figures in other studies, we found that the difference in TSLP expression between the stratum basale and the stratum spinosum was not obvious either [15, 17, 36]. There are two reasons for these discrepancies, namely, a small sample size in both studies and the multiple functions of TSLP. More comprehensive studies with a larger sample size are needed to explain the difference further. The specific expression pattern might reflect the resource of TSLP in the dermatoses. For instance, LP is primarily induced by cytotoxic CD8^+^ T cells attacking the basal keratinocytes, but TSLP is expressed predominantly higher in the spinous keratinocytes. It may imply that the spinous cells play more important roles in pathogenesis of LP than previously thought, whereas in sarcoidosis, TSLP in the skin may come from the dermis or circulation. Moreover, if TSLP was produced from spinous keratinocytes in those dermatoses, it would be valuable to prevent TSLP generation topically. Recently, topical agents were found to reduce TSLP secretion in the epidermis [39, 40]. There is some prospect of lowering TSLP expression in the epidermis, especially the spinous keratinocytes, to improve inflammatory dermatoses.

Finally, this study has potential limitations. As mentioned above, the sample size of this research is small in some extent, whereas our study attempts to conduct a preliminary study and our intention is to raise a concern about TSLP and its effect in cutaneous diseases with different patterns of immune response. We would like to enlarge the sample size in the further study of specific dermatosis. Furthermore, we only detected the levels of TSLP in skin specimens of patients. Some immune-related dermatoses, such as PsV, BP and sarcoidosis, were not only skin diseases but also systemic disorders. It might be meaningful to detect the levels of TSLP in the sera or other samples and to compare the results with those in skin lesions. Moreover, TSLP interacts with IL-33 and other cytokines and mediates a Th2 immune response so that it is valuable to detect other cytokines and compare their expressions [41].

## 5. Conclusions

As a whole, our study found that the expression of TSLP was elevated in different immune response dermatoses. This finding adds to the accumulating evidence of the importance of TSLP in each inflammatory disease. It remains to be elucidated if TSLP plays a role in some inflammatory diseases, such as DLE and sarcoidosis. Tezepelumab, the first-in-class anti-TSLP monoclonal antibody, was approved as a biological agent for the treatment of severe asthma [42]. A randomized phase 2a clinical trial reported that patients of atopic dermatitis have improved under the treatment of tezepelumab [43]. It is necessary to investigate TSLP in those inflammatory diseases further, in order to discover a potential novel therapeutic target.

## Figures and Tables

**Table 1:** Inclusion criteria for specimens used in retrospective analysis.

Disease	Inclusion criteria
LP	Typical clinical manifestation + wedge-shaped hypergranulosis, dense band-like lymphocytic infiltrate with an absence of eosinophils or parakeratosis
DLE	Clinical suspicion + hyperkeratosis, vacuolar interface change, thickened basement membrane with patchy superficial and deep lymphocytic infiltrate
Eczema	Clinical suspicion + spongiosis with superficial perivascular inflammatory infiltrate, and without clear aetiology, not atopic dermatitis or contact dermatitis
BP	Clinical suspicion + subepidermal blister with direct or indirect immunofluorescence and/or autoantibodies
PsV	Clinical suspicion + confluent parakeratosis with regular acanthosis
Sarcoidosis	Clinical suspicion + “naked” granulomas with no/minimal lymphocytes surrounding granuloma, rule tuberculosis and leprosy out
MF	Clinical suspicion + epidermotropic lymphocytes, intraepidermal collections of lymphocytes, atypical lymphocytes, typical immunophenotype with clonal TCR rearrangement

LP: lichen planus; DLE: discoid lupus erythematosus; BP: bullous pemphigoid; PsV: psoriasis vulgaris; MF: mycosis fungoides.

**Table 2:** List of participants for specimens used in a retrospective analysis.

Group	Number of cases	Age, years (mean (range))	Sex	
			Male	Female
LP	10	48.8 (29-65)	3	7
DLE	10	41.5 (24-67)	5	5
Eczema	10	50.2 (27-74)	5	5
BP	10	70.4 (51-87)	5	5
PsV	11	49.7 (26-73)	6	5
Sarcoidosis	10	55.5 (30-76)	3	7
MF	10	38.4 (20-67)	6	4
HC	5	40.8 (28-56)	2	3

Age was record at the time of samples collection. LP: lichen planus; DLE: discoid lupus erythematosus; BP: bullous pemphigoid; PsV: psoriasis vulgaris; MF: mycosis fungoides; HC: healthy control.

**Table 3:** Positive rates of thymic stromal lymphopoietin expression in the epidermis in each group.

Group	Positive rates of TSLP in the stratum basale	Positive rates of TSLP in the stratum spinosum
Total	61.84%	67.11%
LP	70%	60%
DLE	60%	50%
Eczema	50%	70%
BP	70%	80%
PsV	100%	100%
Sarcoidosis	70%	60%
MF	40%	80%
HC	0	0

LP: lichen planus; DLE: discoid lupus erythematosus; BP: bullous pemphigoid; PsV: psoriasis vulgaris; MF: mycosis fungoides; HC: healthy control.

**Table 4:** Pearson's correlation between stratum spinosum and stratum basale thymic stromal lymphopoietin expression.

Group	Correlation coefficient	*p* value
Total	0.713	<0.001
LP	0.654	0.040
DLE	0.673	0.033
Eczema	0.852	0.002
BP	0.904	<0.001
PsV	0.618	0.043
Sarcoidosis	0.886	<0.001
MF	0.305	0.391
HC	-0.250	0.685

LP: lichen planus; DLE: discoid lupus erythematosus; BP: bullous pemphigoid; PsV: psoriasis vulgaris; MF: mycosis fungoides; HC: healthy control.

## Data Availability

The dataset included in this paper is available from the corresponding author on reasonable request and with appropriate additional ethical approvals, when necessary.
